# Rivaroxaban, a specific FXa inhibitor, improved endothelium-dependent relaxation of aortic segments in diabetic mice

**DOI:** 10.1038/s41598-019-47474-0

**Published:** 2019-08-01

**Authors:** Phuong Tran Pham, Daiju Fukuda, Shusuke Yagi, Kenya Kusunose, Hirotsugu Yamada, Takeshi Soeki, Michio Shimabukuro, Masataka Sata

**Affiliations:** 1 Department of Cardiovascular Medicine, Tokushima University Graduate School of Biomedical Sciences, Tokushima, 770-8503 Japan; 2 Department of Cardio-Diabetes Medicine, Tokushima University Graduate School of Biomedical Sciences, Tokushima, 770-8503 Japan; 3 Department of Community Medicine for Cardiology, Tokushima University Graduate School of Biomedical Sciences, Tokushima, 770-8503 Japan; 4 Department of Diabetes, Endocrinology and Metabolism School of Medicine, Fukushima Medical University, Fukushima, 960-1295 Japan

**Keywords:** Experimental models of disease, Vascular diseases, Diabetes complications

## Abstract

Activated factor X (FXa) plays a central role in the coagulation cascade, while it also mediates vascular function through activation of protease-activated receptors (PARs). Here, we examined whether inhibition of FXa by rivaroxaban, a direct FXa inhibitor, attenuates endothelial dysfunction in streptozotocin (STZ)-induced diabetic mice. Induction of diabetes increased the expression of a major FXa receptor, PAR2, in the aorta (*P* < 0.05). Administration of rivaroxaban (10 mg/kg/day) to diabetic wild-type (WT) mice for 3 weeks attenuated endothelial dysfunction as determined by acetylcholine-dependent vasodilation compared with the control (*P* < 0.001), without alteration of blood glucose level. Rivaroxaban promoted eNOS^Ser1177^ phosphorylation in the aorta (*P* < 0.001). Induction of diabetes to PAR2-deficient (PAR2^−/−^) mice did not affect endothelial function and eNOS^Ser1177^ phosphorylation in the aorta compared with non-diabetic PAR2^−/−^ mice. FXa or a PAR2 agonist significantly impaired endothelial function in aortic rings obtained from WT mice, but not in those from PAR2^−/−^ mice. FXa promoted JNK phosphorylation (*P* < 0.01) and reduced eNOS^Ser1177^ phosphorylation (*P* < 0.05) in human coronary artery endothelial cells (HCAEC). FXa-induced endothelial dysfunction in aortic rings (*P* < 0.001) and eNOS^Ser1177^ phosphorylation (*P* < 0.05) in HCAEC were partially ameliorated by a JNK inhibitor. Rivaroxaban ameliorated diabetes-induced endothelial dysfunction. Our results suggest that FXa or PAR2 is a potential therapeutic target.

## Introduction

Accumulating evidence suggests that the blood coagulation system participates in various disease processes. Activated blood coagulation factor X (FXa) plays a central role in the coagulation cascade, while it also elicits various cellular responses in vascular cells through activation of protease activated receptors (PAR)^1–5^. PARs are a family of G protein-coupled, seven transmembrane domain receptors, which are activated following proteolytic cleavage of their extracellular N-terminus. Among the four subtypes of the PAR family, PAR2, one of the major receptors of FXa, is expressed in vascular cells and leukocytes, but not in platelets^6–8^. Previous studies have shown that PAR2 mediates a number of physiological and pathological processes such as inflammation, angiogenesis and vasoregulation^9–14^. Together with the clinical approval of direct FXa inhibitors, the role of FXa-PAR2 signaling in vascular regulation has attracted much attention. In fact, several clinical studies have shown that rivaroxaban, the first oral FXa inhibitor, reduced cardiovascular events in patients with coronary artery disease^15,16^. In addition to inhibition of the coagulation cascade, several basic research studies, including our own, suggested that vascular protective properties of rivaroxaban by inhibiting FXa might play a role in these results^17–21^.

The vascular complications associated with atherosclerosis are the most serious manifestations in patients with diabetes. Diabetes causes endothelial dysfunction characterized with increased platelet aggregation, coagulation, and vascular tone, all of which associate with the initiation of atherosclerosis^22^. Numerous studies have demonstrated that hyperglycemia leads to an impairment of NO production and activity in patients with diabetes, leading to the development of endothelial dysfunction^22–25^. Although multifactorial in etiology^26^, recent studies suggested involvement of coagulation factors in the regulation of endothelial function^27^. However, few studies have examined the role of FXa-PAR2 signaling in the development of endothelial dysfunction in a diabetic condition^28–30^. We, here, hypothesized that inhibition of FXa by rivaroxaban attenuates the development of endothelial dysfunction in a diabetic condition. To address this hypothesis, we administered rivaroxaban to diabetic wild-type (WT) mice, and examined vascular responses. We also examined vascular responses of aortic rings obtained from diabetic PAR2-deficient (PAR2^−/−^) mice, and performed *in vitro* experiments using human coronary artery endothelial cells (HCAEC) treated with FXa or a PAR2 agonist to investigate the underlying mechanisms. The results of our experiments indicated that rivaroxaban ameliorated diabetes-induced endothelial dysfunction and suggested that the inhibition of FXa or PAR2 contributes to this disease context.

## Results

### Expression of PAR2 in aorta increased in diabetic mice

**Figure 1:**
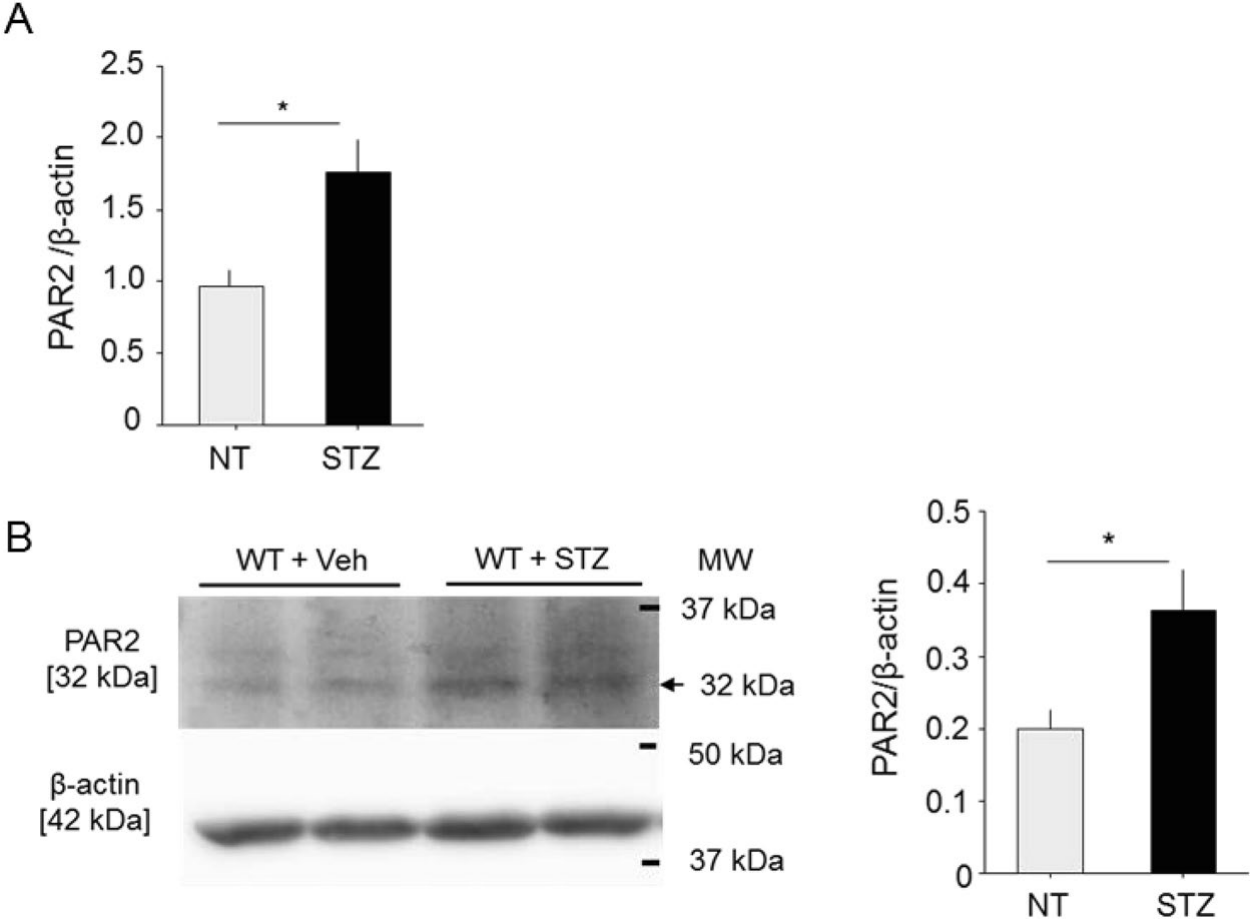
Induction of diabetes increased expression of PAR2 in aorta. (**A**) qPCR analysis using thoracic aorta demonstrated that induction of diabetes by STZ increased the expression of PAR2 (n = 6). **(B)** Western blot data demonstrated that induction of diabetes increased the expression of PAR2 in the aorta (n = 8–10). *P < 0.05. All values are mean ± SEM.

We examined the expression of PAR2, a major receptor for FXa, in the thoracic aorta of diabetic WT mice. Diabetic mice had significantly higher expression of PAR2 in both RNA and protein levels compared with the non-diabetic group (Fig. 1A,B). These results indicated elevation of PAR2 expression under a hyperglycemic condition.

### Rivaroxaban improved endothelial dysfunction in WT diabetic mice

**Figure 2:**
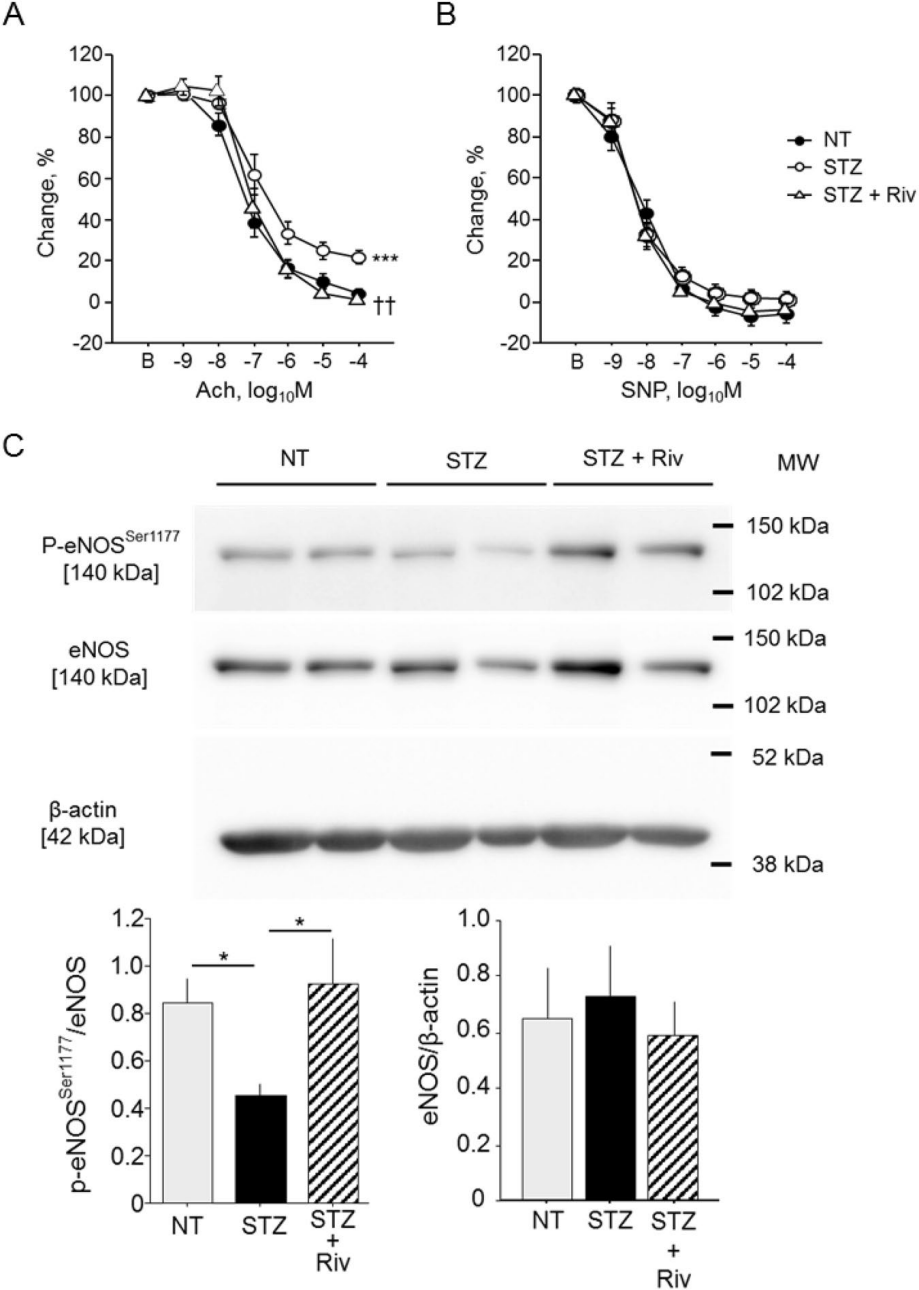
Rivaroxaban attenuated endothelial dysfunction in diabetic mice. (**A,B**) Induction of diabetes by STZ impaired endothelium-dependent vasodilation in response to Ach compared with the non-diabetes group. Rivaroxaban administration to diabetic mice for 3 weeks ameliorated endothelial function compared with the non-treated group (**A**). Vasorelaxation in response to SNP did not differ among the three groups (**B**) (n = 8–12). ^***^P < 0.001 vs. NT, and ^††^*P* < 0.01 vs. STZ. (**C**) Induction of diabetes attenuated eNOS phosphorylation at Ser1177; however, rivaroxaban treatment ameliorated this response (n = 10–13). There was no significant difference in total eNOS expression among 3 groups. ^*^P < 0.05. All values are mean ± SEM.

We next investigated whether rivaroxaban ameliorates endothelial dysfunction in WT diabetic mice. Induction of diabetes impaired endothelium-dependent vasodilation in response to acetylcholine (Ach) (*P* < 0.001). However, rivaroxaban treatment for 3 weeks significantly attenuated impairment of endothelial function in diabetic mice compared with non-treated mice (*P* < 0.01) (Fig. 2A). In contrast, endothelium-independent vascular response to sodium nitroprusside (SNP) did not differ between rivaroxaban-treated mice and non-treated mice (Fig. 2B). Administration of rivaroxaban for 3 weeks did not affect blood glucose level or total cholesterol level, although it reduced triglyceride level in diabetic WT mice (Table 1). Consistent with the results of vascular reactivity analyses, the results of western blotting using abdominal aorta showed that phosphorylation of eNOS^Ser1177^ was significantly promoted in the rivaroxaban-treated group compared with the non-treated group (*P* < 0.05) (Fig. 2C). These results suggest that rivaroxaban, a direct FXa inhibitor, attenuates endothelial dysfunction in diabetic WT mice.

**Table 1:** Effect of rivaroxaban on metabolic parameters.

	NT (n = 12)	STZ (n = 13)	STZ + Riv (n = 10)	P-value
Body weight, g	26.5 ± 0.8	17.5 ± 0.5^***^	16.9 ± 0.5^***^	<0.001
Blood glucose, mg/dl	166.8 ± 5.3	641.5 ± 27.6^***^	607.1 ± 23.4^***^	<0.001
Total cholesterol, mg/dl	115.8 ± 2.0	358.9 ± 17.0^***^	441.8 ± 78.4^***^	<0.001
Triglyceride, mg/dl	137.1 ± 12.3	346.2 ± 51.8	133.2 ± 55.6^††^	<0.01
HDL-cholesterol, mg/dl	75.5 ± 1.9	161.5 ± 4.2^***^	145 ± 12.9***	<0.001

NT; non-treatment, STZ; streptozotocin, Riv; rivaroxaban, HDL; high-density lipoprotein.

***P < 0.001 vs. NT, and ^††^P < 0.01 vs. STZ.

### FXa induced endothelial dysfunction via JNK pathway

**Figure 3:**
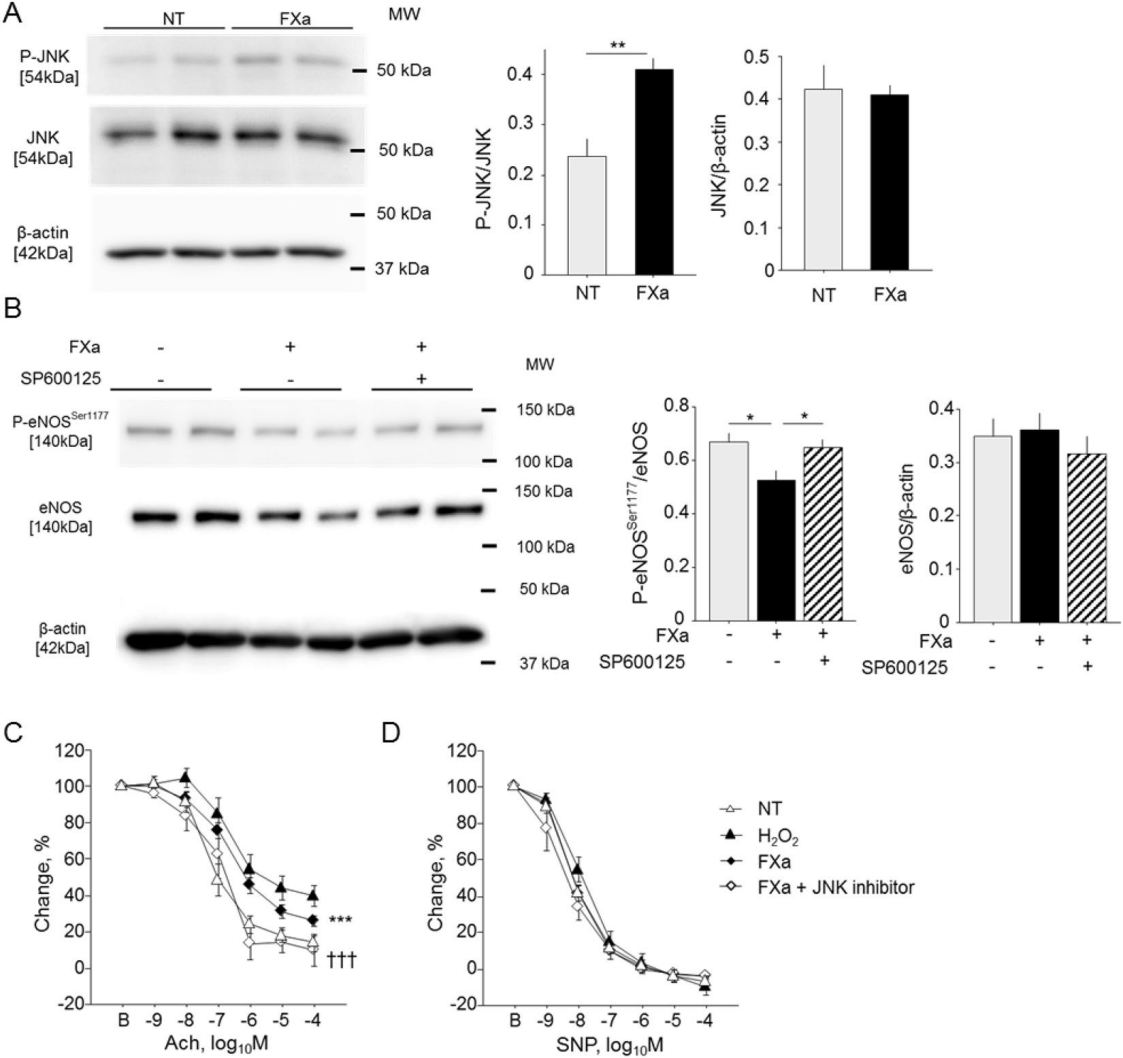
JNK inhibitor attenuated endothelial dysfunction induced by FXa. (**A**) FXa promoted the phosphorylation of JNK in HCAEC (n = 9). **P < 0.01. (**B**) FXa attenuated the phosphorylation of eNOS at Ser1177 in HCAEC. Pretreatment with SP600125, a JNK inhibitor, ameliorated this response (n = 9). ^*^P < 0.05. (**C,D**) Vascular reactivity in response to Ach (**C**) or SNP (**D**) was examined using aortic segments obtained from WT mice (n = 5–12). FXa impaired endothelium-dependent vasodilation in response to Ach. SP600125 attenuated endothelial dysfunction induced by FXa. H_2_O_2_ was used for showing the impaired endothelial dysfunction. Vasorelaxation in response to SNP did not differ in the presence of FXa or SP600125. ^***^P < 0.001 vs. NT, and ^†††^*P* < 0.001 vs. FXa. All values are mean ± SEM.

Rivaroxaban is an inhibitor of FXa. To investigate the effect of FXa on endothelial function, we performed western blot analysis using HCAEC. FXa promoted phosphorylation of JNK in HCAEC (*P* < 0.01) (Fig. 3A). FXa reduced phosphorylation of eNOS^Ser1177^ (*P* < 0.05), which was attenuated by SP600125, a JNK inhibitor (*P* < 0.05) in this cell-type (Fig. 3B). We further performed *ex vivo* experiments using aortic rings obtained from WT mice. FXa significantly impaired endothelium-dependent vascular reactivity in response to Ach (*P* < 0.001), while SP600125 ameliorated FXa-induced endothelial dysfunction (*P* < 0.001) (Fig. 3C). Neither FXa nor SP600125 affected endothelium-independent vasodilation in response to SNP (Fig. 3D). These results suggest that FXa induced endothelial dysfunction, at least partially, via the JNK pathway.

### FXa and PAR2 is associated with endothelial dysfunction in diabetic condition

**Figure 4:**
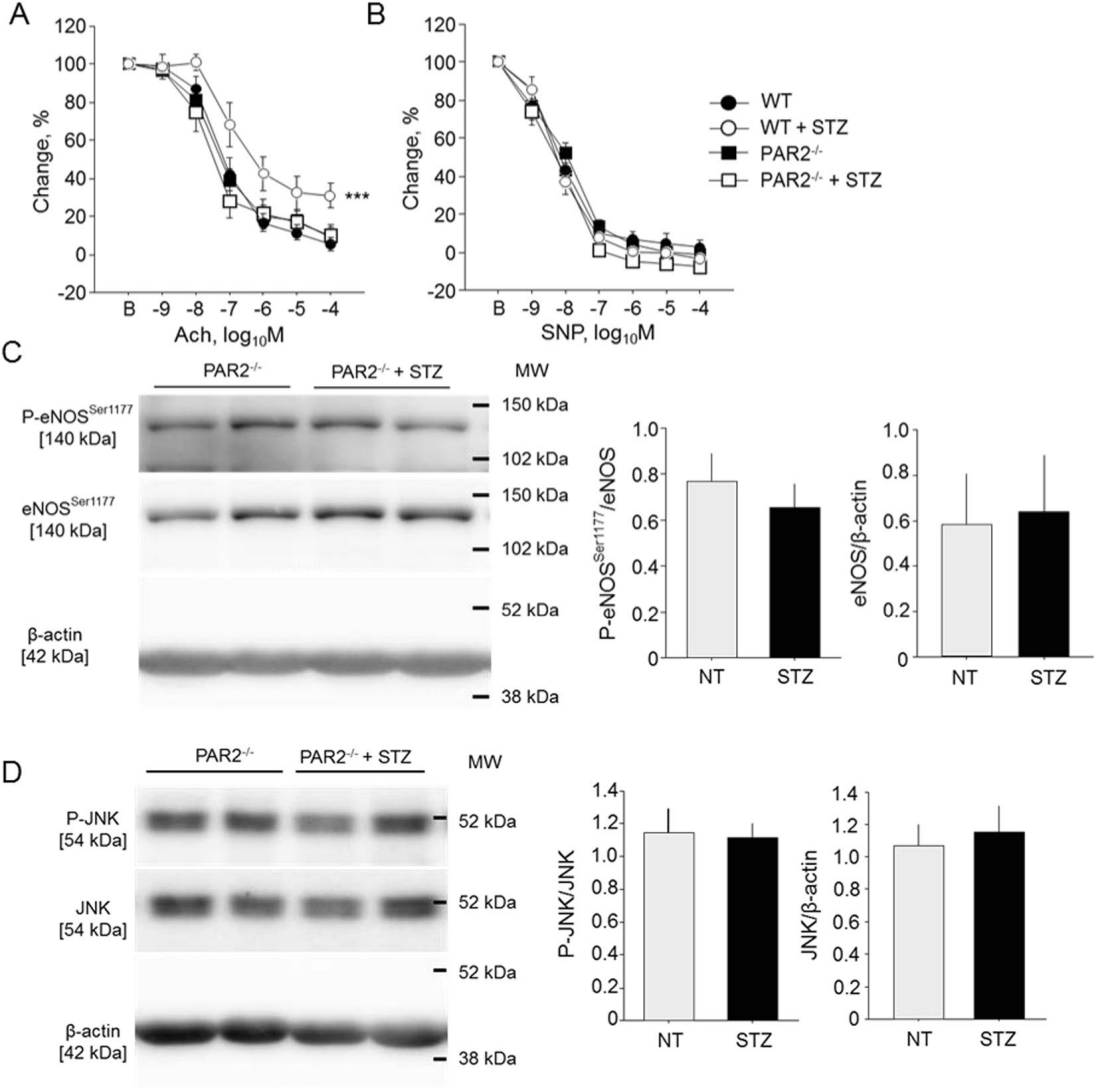
Genetic deletion of PAR2 prevented endothelial dysfunction caused by diabetes. (**A,B**) Vascular reactivity in response to Ach (**A**) or SNP (**B**) was examined using aortic segments obtained from diabetic or non-diabetic mice (n = 8–14). Induction of diabetes impaired endothelial function determined by the response to Ach in WT mice. Induction of diabetes to PAR2^−/−^ mice did not affect vascular response to Ach or SNP. ***P < 0.001 vs. non-diabetic WT mice. **(C**,**D)** Induction of diabetes in PAR2^−/−^ mice did not affect the phosphorylation of eNOS^Ser1177^ (**C**) and JNK (**D**) (n = 6). There was no significant difference in total eNOS and JNK expression between 2 groups. All values are mean ± SEM.

**Figure 5:**
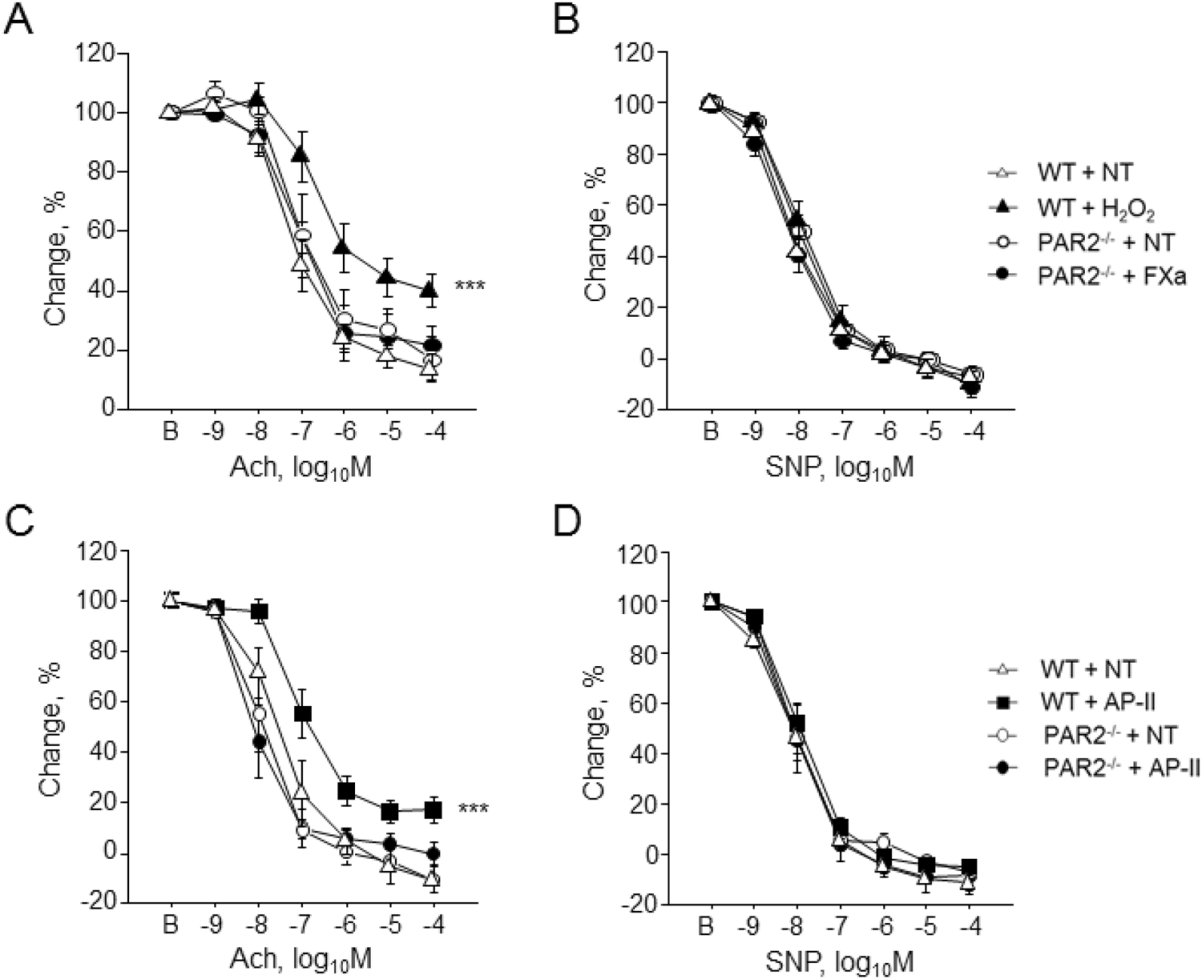
Activation of PAR2 impaired endothelial function. (**A**,**B**) Vascular reactivity in response to Ach (**A**) or SNP (**B**) was examined using aortic segments obtained from WT mice or PAR2^−/−^ mice (n = 6–11). FXa did not impair endothelium-dependent (**A**) and -independent (**B**) vascular response in aortic segments obtained from PAR2^−/−^ mice. H_2_O_2_ was used for showing the impaired endothelial dysfunction in WT mice. ***P < 0.001 vs. WT + NT. (**C,D**) Vascular reactivity to AP-II, a specific agonist for PAR2, was determined using aortic segments isolated from WT and PAR2^−/−^ mice (n = 5–7). AP-II impaired endothelium-dependent vasodilation in response to Ach in aortic segments obtained from WT mice. However, AP-II did not affect endothelial function in aortic segments obtained from PAR2^−/−^ mice (**C**). AP-II did not affect vasorelaxation in response to SNP in aortic segments obtained from both strains of mouse (D). ***P < 0.001 vs. WT + NT. All values are mean ± SEM.

**Figure 6:**
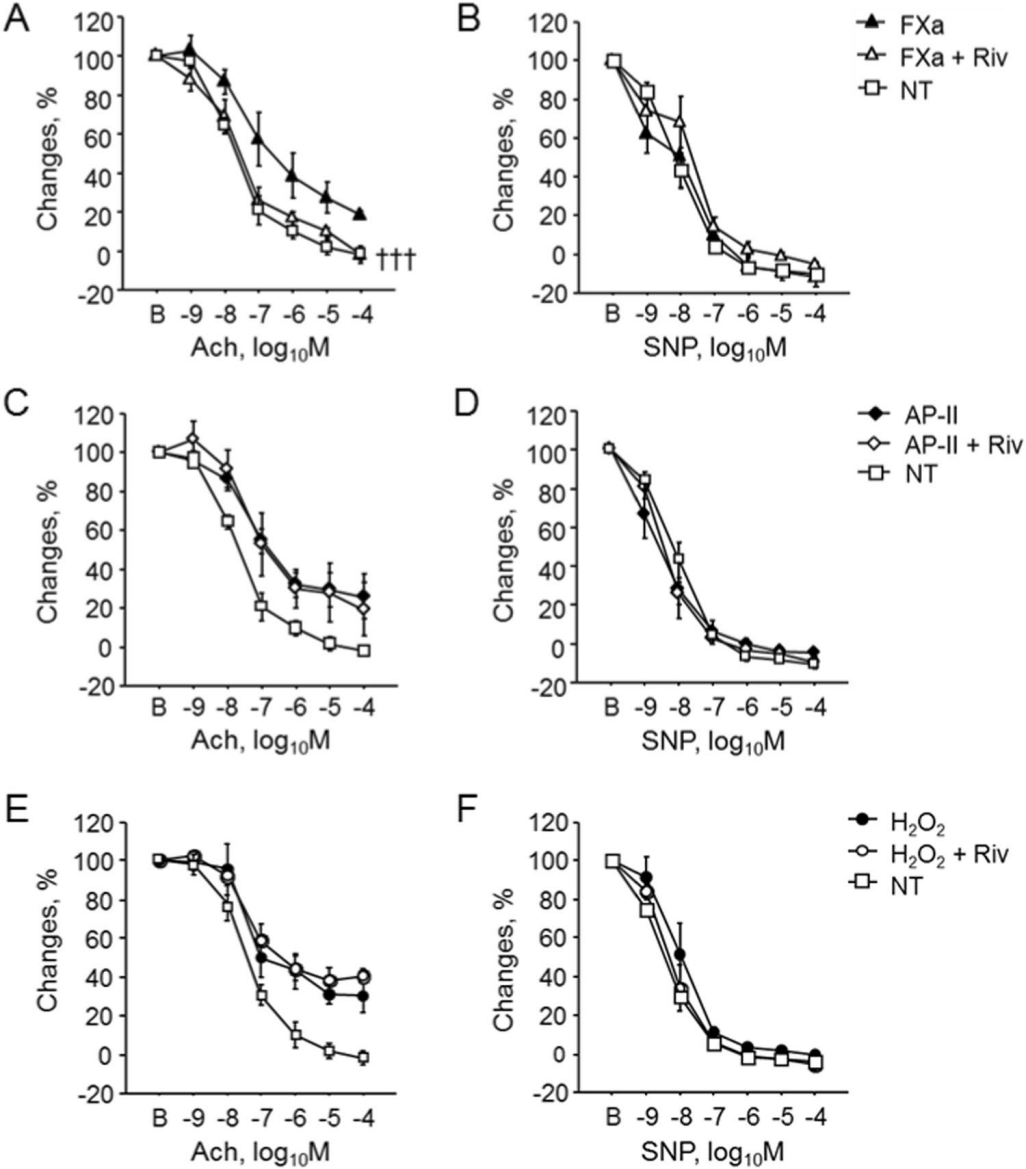
Rivaroxaban ameliorated FXa-induced endothelial dysfunction. Rivaroxaban ameliorated FXa-induced endothelial dysfunction (**A**), although it did not have effects on endothelial dysfunction induced by AP-II (**C**) or H_2_O_2_ (**E**). Rivaroxaban did not affect endothelial-independent vascular response induced by FXa (**B**), AP-II (**D**), or H_2_O_2_ (**F**) (n = 4–7). ^†††^*P* < 0.001 vs. FXa. All values are mean ± SEM.

PAR2 is a major receptor of FXa. Therefore, to investigate the role of PAR2 in the development of endothelial dysfunction in diabetic mice, we induced diabetes in WT and PAR2^−/−^ mice by STZ injection and examined vascular response. Induction of diabetes significantly impaired endothelial function as determined by Ach-dependent vasodilation in WT mice but not in PAR2^−/−^ mice (Fig. 4A). STZ injection elevated blood glucose and lipid levels in both strains of mouse; however, there were no significant differences between non-diabetic WT mice and PAR2^−/−^ mice or between diabetic WT mice and PAR2^−/−^ mice (Table 2). Endothelium-independent vascular response to SNP did not differ between groups (Fig. 4B). The results of western blotting using abdominal aorta demonstrated no significant differences in the phosphorylation of eNOS^Ser1177^ and JNK between diabetic and non-diabetic PAR2^−/−^ mice (Fig. 4C,D). We also examined the role of PAR2 in endothelial function. FXa impaired endothelial function of aortic rings from WT mice (Fig. 3C); however, this response was not observed in aortic rings from PAR2^−/−^ mice (Fig. 5A). FXa did not affect endothelium-independent vasodilation (Fig. 5B). We further examined the effect of pharmacological activation of PAR2 using a specific PAR2 agonist, AP-II. Incubation with AP-II significantly impaired endothelium-dependent vasodilation of aortic rings from WT mice (*P* < 0.001) but not of aortic rings from PAR2^−/−^ mice (Fig. 5C). AP-II did not affect endothelium-independent vasodilation of aortic rings from both strains of mouse (Fig. 5D). In addition, we examined effects of rivaroxaban on FXa-induced endothelial dysfunction. Rivaroxaban ameliorated FXa-induced endothelial dysfunction. Rivaroxaban did not have effects on endothelial dysfunction induced by AP-II or H_2_O_2_ (Fig. 6). These results suggest that FXa-PAR2 signaling contributes, at least partially, to the development of endothelial dysfunction.

**Table 2:** Effect of STZ injection on metabolic parameters.

	WT		PAR2^−/−^	
	NT (n = 14)	STZ (n = 9)	NT (n = 9)	STZ (n = 8)
Body weight, gram	26.2 ± 0.4	17.3 ± 0.7***	26.2 ± 1.2	17.3 ± 0.5***
Blood glucose, mg/dl	151.4 ± 6.8	630.4 ± 28.1***	135.2 ± 8.5	507.8 ± 29.7***
Total cholesterol, mg/dl	110.2 ± 3.0	439.9 ± 88.3***	117.6 ± 3.5	417.1 ± 33.7***
Triglyceride, mg/dl	114.9 ± 8.9	403.1 ± 110.3^**^	141.3 ± 30.5	461.5 ± 100.5^**^
HDL-Cholesterol, mg/dl	67.7 ± 1.9	149.8 ± 15.3***	74.6 ± 2.3	153.1 ± 7.6***

WT; wild-type, NT; non-treatment, STZ; streptozotocin,HDL; high-density lipoprotein.

^*^P < 0.05, ^**^P < 0.01, and ***P < 0.001 vs. NT.

## Discussion

Accumulating evidence suggests that FXa-PAR2 signaling contributes to various pathological conditions^31–34^. In this study, we found that rivaroxaban, a FXa inhibitor, significantly attenuated endothelial dysfunction in diabetic WT mice, with no alteration of blood glucose level. On the other hand, FXa or AP-II impaired endothelium-dependent vasodilation of aortic segments obtained from WT mice. Furthermore, rivaroxaban ameliorated endothelial dysfunction induced by FXa but not AP-II. In addition, induction of diabetes in PAR2^−/−^ mice did not impair endothelial function. Also, neither FXa nor AP-II affected endothelial function of aortic segments obtained from PAR2^−/−^ mice. The results of *in vitro* experiments using HCAEC indicated that FXa causes endothelial dysfunction as determined by eNOS^Ser1177^ phosphorylation, partially through JNK signaling, supporting the *in vivo* results. These results indicated that rivaroxaban ameliorates diabetes-related endothelial dysfunction and suggested that the inhibition of FXa or PAR2 plays a role as an underlying mechanism at least partially.

Rivaroxaban is the first oral anticoagulant that directly inhibits FXa and suppresses thrombin formation. Thrombin plays a pivotal role not only in the blood coagulation cascade, but also in vasoregulation by activating multiple cell types in the vasculature such as endothelial cells, smooth muscle cells, and macrophages. Recent clinical trials demonstrated that patients with coronary artery disease treated with rivaroxaban experienced fewer cardiovascular events compared with the control^15,16^. The anti-coagulation effects of rivaroxaban might play a role in these results, although these studies also suggest that the inhibition of FXa by rivaroxaban might improve vascular function.

FXa activates PAR1 and PAR2^6–8^. Because of its unique expression pattern, previous studies investigated the role of PAR2 in vascular function^17,19,20,35^. We and others have demonstrated that inhibition of FXa-PAR2 signaling by FXa inhibitors including rivaroxaban or genetic deletion of PAR2 attenuates atherogenesis in atherosclerotic mouse models^17,20^. Endothelial function is essential for homeostasis of the vasculature, and its impairment initiates atherosclerotic changes^36^. In diabetic patients, endothelial function was impaired by one or a combination of factors such as hyperglycemia, fatty acids, inflammation, and insulin resistance. In parallel, previous studies indicated that endothelial dysfunction is reversible and a potential therapeutic target in atherosclerotic disease^37,38^. Therefore, in this study, we investigated the effect of rivaroxaban on diabetes-related endothelial dysfunction. Previous studies have shown vasoprotective effects of rivaroxaban. Rivaroxaban enhanced viability, growth and migration of endothelial cells, suggesting its protective effects on endothelium^39^. Rivaroxaban also decreased FXa-induced cell senescence of endothelial cells and endothelial progenitor cells, accelerating neovascularization in the ischemic limb^40^. Several previous studies using diabetic mouse models demonstrated that rivaroxaban promoted vessel formation^41^, and that a PAR2 antagonist reduced endothelial dysfunction^42^. In this study, we demonstrated that both rivaroxaban, a clinically approved FXa inhibitor, and genetic deletion of PAR2 clearly attenuated the development of endothelial dysfunction in diabetic mice, without alteration of blood glucose level. On the other hand, FXa or AP-II attenuated endothelial function of aortic rings, supporting our *in vivo* results. In addition, our *in vitro* experiments using HCAEC demonstrated that FXa reduced phosphorylation of eNOS^Ser1177^ partially through JNK activation. In fact, a JNK inhibitor ameliorated FXa-induced impairment of endothelium-dependent vascular response. JNK activation is essential for endothelial dysfunction by inhibiting eNOS^Ser1177^ phosphorylation^43,44^. Previous studies showed that PAR2 activation enhances the phosphorylation of JNK in several cell types including endothelial cells^32,45–47^. Thus, our study indicates the involvement of FXa-PAR2 signaling, at least partially, as an underlying mechanism in diabetes-related endothelial dysfunction. The signaling pathways associated with PARs are not fully understood. Therefore, further studies are required to elucidate the precise mechanism by which FXa-PAR2 signaling causes endothelial dysfunction.

Recent studies including our present study showed that inhibition of FXa-PAR2 signaling has protective effects on the endothelium. However, several previous studies have demonstrated protective roles of PAR2 in vascular function. For example, studies using a septic shock model showed protective roles of FXa in vascular inflammation and vascular response^48,49^. Other studies demonstrated the angiogenic potential of PAR2^11,50^. Furthermore, one study demonstrated that PAR2 mediates arterial vasodilation in diabetes^51^. Because previous studies suggested that the function or signaling of PAR2 may vary depending on disease context^14,52^, the differences in mouse model and strategy for PAR2 inhibition may explain the discrepancy among these studies. Dissecting the role of FXa-PAR2 signaling in vascular function and its signaling mechanisms requires further studies.

There are several limitations of our study. First, we did not examine the plasma concentration of rivaroxaban in this study. However, we did not observe any bleeding complication throughout the study period in the treated mice. Secondly, FXa activates PAR1 as well as PAR2, although we focused only on the role of PAR2 in this study. PAR1 might contribute to the result of this study. Also, we used systemic deletion model of PAR2 in this study. Smooth muscle cells and leukocytes express PAR2^6,7^. Therefore, the activation of PAR2 on these cell types might participate in our results. Thirdly, we observed a reduction of triglyceride by the administration of rivaroxaban in STZ-induced diabetic WT mice. In our previous studies, rivaroxaban did not reduce triglyceride level in WT mice and apolipoprotein e-deficient mice. Triglyceride level in STZ-induced diabetic WT mice was shown to be higher than that in non-diabetic mice in other studies^53^. The results of the present study might be because of the mouse model we used. Further studies are needed in relation to this. Fourthly, we did not examine dose dependent effects and effects of other FXa inhibitor. These investigations would provide stronger evidence for the effectiveness of rivaroxaban or FXa inhibition on diabetes-induced endothelial dysfunction. Last, the results of our present study were obtained from only basic studies. Therefore, the results of our study might not applied to clinical situation directly. On the other hand, we showed that clinically approved rivaroxaban, a FXa inhibitor, inhibited the development of diabetes-induced endothelial dysfunction. Together with our previous studies that reported atheroprotective effects of rivaroxaban, the results of our present study might explain one of the mechanisms for recent clinical studies that reported fewer cardiovascular events in patients with coronary artery disease treated with rivaroxaban compared with the control^15,16^. Also, we used not only pharmacological blockade of FXa by using rivaroxaban but also PAR2 deficient mouse model or *ex vivo* vascular reactivity experiments using AP-II. These experiments allowed us to examine the effects of FXa or PAR2 from multiple aspects. We thought that these are the strength of our present study.

In conclusion, our study demonstrated that rivaroxaban attenuated the development of endothelial dysfunction in diabetic mice, independently of blood glucose level, possibly through partial inhibition of FXa-PAR2 signaling. Rivaroxaban may provide a therapeutic option for diabetes-related endothelial dysfunction, and FXa and/or PAR2 might be a potential therapeutic target for this disease condition.

## Materials and Methods

### Animal and drug administration

C57BL/6J wild-type mice and PAR2^−/−^ mice (C57BL/6J background) were purchased from Japan SLC, Inc. and Jackson Laboratory, respectively. Diabetes was induced by a single intraperitoneal injection of streptozotocin (SZT, 180 mg/kg) to 8-week-old male mice after overnight fasting. The non-diabetic control group received an injection of vehicle (0.1 M sodium citrate). Rivaroxaban (10 mg/kg/day) supplemented to normal chow was administered for 3 weeks, immediately after STZ injection. The non-treated group received non-supplemented chow. Rivaroxaban was supplied by Bayer Pharma AG. Mice were housed in a room in which the temperature was kept at 23 °C and the light cycle was automatically controlled (12 hours dark/light). All procedures for animal experiments conformed to the guidelines for animal experimentation of Tokushima University. The protocol was reviewed and approved by the Animal Care and Use Committee of Tokushima University under #T29-113.

### Blood glucose level and laboratory data

Glucose levels in tail vein blood were measured using Startstrip XP2 (NIPRO) at three time points: before fasting, day 3 after injection to confirm a diabetic condition, and at the time of harvest. Blood was collected from the left ventricle into EDTA-containing tubes. Plasma was separated from whole blood at 9000 rpm for 15 minutes at 4 °C and kept at −80 °C until required. Plasma lipid levels (e.g., total cholesterol, HDL-cholesterol, and triglyceride) were measured at LSI Medience Corporation (Japan).

### Aorta preparation and vascular reactivity assay

At 3 weeks after the initiation of rivaroxaban administration, mice were sacrificed by an overdose of pentobarbital, and perfused with 0.9% sodium chloride solution via the left ventricle at constant pressure. The whole aorta was immediately isolated, and fat and connective tissue around the aorta were carefully removed. Aortic rings of 1.5 to 2 mm were cut from the thoracic aorta for vascular reactivity analysis. The remaining part of the thoracic aorta and the abdominal aorta were snap-frozen in liquid nitrogen for gene expression or western blotting analysis, respectively.

Vascular reactivity was examined as described previously^54^. Aortic rings were placed in organ baths filled modified Krebs-Henseleit buffer (118.4 mM NaCl, 4.7 mM KCl, 2.5 mM CaCl_2_, 1.2 mM KH_2_PO_4_, 1.2 mM MgSO_4_, 25 mM NaHCO_3_, 11.1 mM glucose) that was aerated (95% O_2_, 5% CO_2_) and warmed (37 °C). Changes in isometric tension were recorded on a polygraph (LabChart). The viability of aortic segments was tested with 31.4 mM KCl. To determine the relaxation response, the aortic rings were contracted with phenylephrine (10^−9^ to 10^−4^ M) to submaximal tension (60% of maximum). After stable contraction was determined, the rings were exposed to increasing concentrations of acetylcholine (Ach; 10^−9^ to 10^−4^ M) and SNP (10^−9^ to 10^−4^ M) to obtain a cumulative concentration-response curve. In *ex-vivo* experiments, aortic segments were pre-treated with 100 nM SP600126 (Sigma Aldrich), a JNK inhibitor, for 2 hours, and then incubated with 10 nM mouse FXa (Heamatologic Technologies, Inc.) for 4 hours before analyses of vascular reactivity. To investigate the role of PAR2 in vascular function, aortic segments were incubated with 10 nM AP-II (Sigma Aldrich), a PAR-2 agonist, for 16 hours. In some experiments, H_2_O_2_ was used for the induction of endothelial dysfunction. To investigate the effect of rivaroxaban on FXa or AP-II, we pre-treated with aortic rings with 1 μM rivaroxaban for 16 hours.

### Cell culture

HCAEC were purchased from Lifeline cell Technology and cultured in EGM-2 (Lonza) in a 5% CO_2_ humidified atmosphere. To investigate the effect of FXa on endothelial cells, HCAEC (passages 4-6) were treated with 10 nM human Factor X (Heamatologic Technologies, Inc.) in EBM-2 (Lonza) containing 2% FBS for 1 hour. In addition, to block the JNK pathway, HCAEC were pre-treated with 100 nM SP600125 for 2 hours, and then stimulated with human FXa for 1 hour.

### Western blotting analysis

Total protein was exacted from cells or aortic tissue using RIPA buffer (Wako Pure Chemical Industries, Ltd.) containing a protease inhibitor cocktail (Roche) and phosphatase inhibitors (Roche). The concentration of protein was determined using a BCA protein assay kit (Thermo Scientific). Equal quantities of protein (10 μg) were separated using 7.5% sulfate–polyacrylamide gel and transferred onto polyvinylidene difluoride membranes. After blocking with 5% bovine serum albumin, the membranes were incubated with a primary antibody; anti-phosphorylated eNOS^Ser1177^, anti-eNOS (BD Biosciences), anti-phosphorylated JNK, or anti-JNK (Cell Signaling Technology), overnight at 4 °C. For detection of β-actin, the membranes were blocked with 5% skimmed milk and incubated with anti-β-actin antibody (Sigma) for 1 hour at room temperature. HRP-conjugated anti-mouse Ig or anti-rabbit Ig was used as a second antibody. Immunoblotting was analyzed with ECL-plus reagent (GE Healthcare) using a luminescent image analyzer (LAS-4000 mini, Fuji Film).

### Quantitative Real-time PCR

An Illustra RNAspin RNA Isolation Kit (GE Healthcare) was used to extract total RNA from tissues. A QuantiTecht Reverse Transcription Kit (Qiagen) was used for synthesis of cDNA from 100 ng RNA. Mx3000P (Agilent Technologies) and Power SYBR Green PCR master mix (Applied Biosystems) were used for quantitative real-time PCR. The following oligonucleotide primers were used: PAR2, forward: 5′-GGACCGAGAACCTTGCAC-3′ and reverse: 5′-GAACCCCTTTCCCAGTGATT-3′ and β-actin: forward: 5′-CCTGAGCGCAAGTACTCTGTGT-3′ and reverse: 5′-GCTGATCCACATCTGCTGGAA-3′. β-actin was used as the reference gene.

### Statistical analyses

All data were presented as mean ± SEM. Comparison of parameters between two groups was performed with unpaired Student’s t-test when data showed a normal distribution or with Mann-Whitney U test when data did not show a normal distribution. Differences between multiple groups were analyzed by ANOVA followed by Tukey’s post hoc analysis when data showed a normal distribution or with Kruskal–Wallis test followed by Dunn’s post hoc analysis when data did not show a normal distribution. Comparison of dose-response curves was performed by two-factor repeated measures ANOVA, followed by Tukey’s post hoc test for comparison between groups. *P* < 0.05 was considered statistically significant.

## Supplementary information

Supplementary Figure 1-6

## Data Availability

The datasets generated during and/or analyzed during the current study are available from the corresponding author on reasonable request.
