# Supplementary material for: Rivaroxaban, a specific FXa inhibitor, improved endothelium-dependent relaxation of aortic segments in diabetic mice
Source: Sci Rep. 2019 Aug 1;9:11206. doi: 10.1038/s41598-019-47474-0 (PMC6672013; doi:10.1038/s41598-019-47474-0)
Supplement: Supplementary file 1 — Supplementary Figure 1-6 [file 41598_2019_47474_MOESM1_ESM.docx]

**Supplementary Material**

**Rivaroxaban, a specific FXa inhibitor, improved endothelium-dependent relaxation of aortic segments in diabetic mice**

Phuong Tran Pham^1^, Daiju Fukuda^2^, Shusuke Yagi^1^, Kenya Kusunose^1^, Hirotsugu Yamada^3^, Takeshi Soeki^1^, Michio Shimabukuro^2,4^, Masataka Sata^1^

1. Department of Cardiovascular Medicine, Tokushima University Graduate School of Biomedical Sciences, Tokushima 770-8503, Japan.
2. Department of Cardio-Diabetes Medicine, Tokushima University Graduate School of Biomedical Sciences, Tokushima 770-8503, Japan.
3. Department of Community Medicine for Cardiology, Tokushima University Graduate School of Biomedical Sciences, Tokushima 770-8503, Japan.
4. Department of Diabetes, Endocrinology and Metabolism School of Medicine, Fukushima Medical University, Fukushima 960-1295, Japan

Short title; FXa-PAR2 signal in diabetic endothelial dysfunction

Supplementary Figures; 6 figures

All correspondence should be addressed to:

Daiju Fukuda, MD, PhD

Department of Cardiovascular Medicine,

Tokushima University Graduate School of Biomedical Sciences

3-18-15, Kuramoto-cho, Tokushima 770-8503, Japan

Phone: +81-88-633-7859, Fax: +81-88-633-7894

E-mail: [daiju.fukuda@tokushima-u.ac.jp](mailto:daiju.fukuda@tokushima-u.ac.jp)


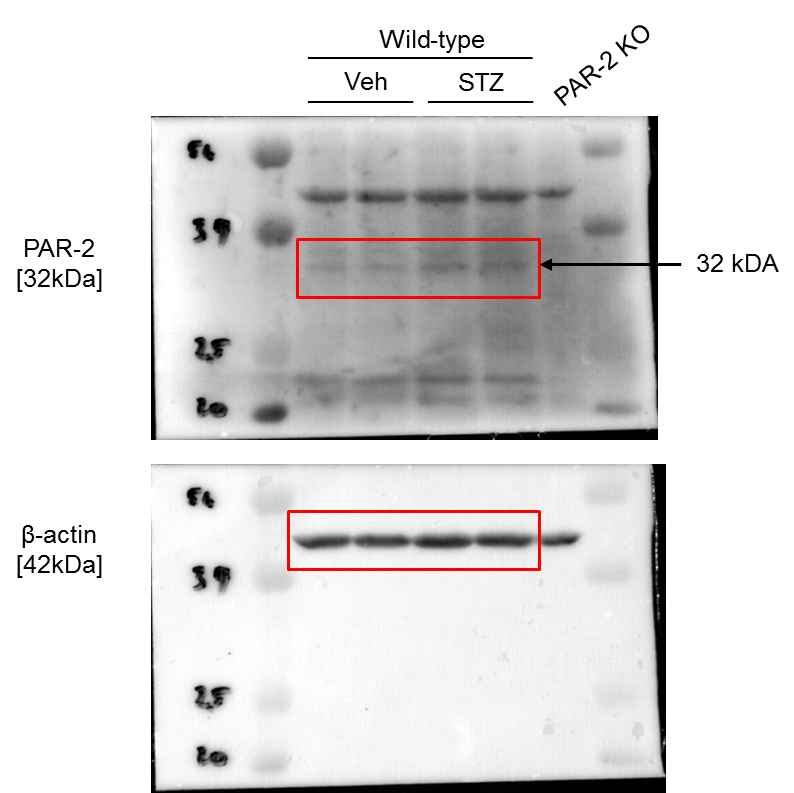


**Supplementary Figure 1. Full-length blot of Figure 1B**

The original full-length immunoblots related to Figure 1B are presented. The upper membrane was incubated with anti-PAR2 antibody. The lower membrane was incubated with anti-β-actin antibody.

**
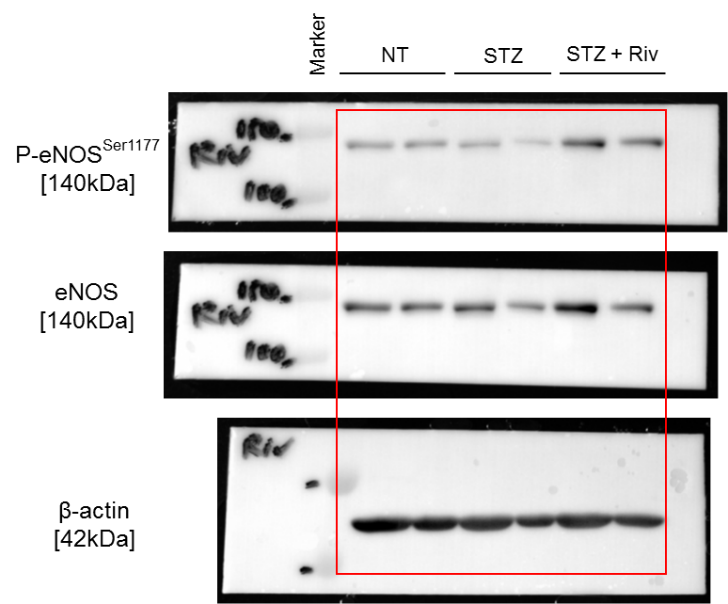
**

**Supplementary Figure 2. Full-length blot of Figure 2C**

The original full-length immunoblots related to Figure 2C are presented. The top membrane was incubated with anti-phosphorylated eNOS^Ser1177^ antibody. The middle membrane was incubated with anti-total eNOS antibody. The bottom membrane was incubated with anti-β-actin antibody.


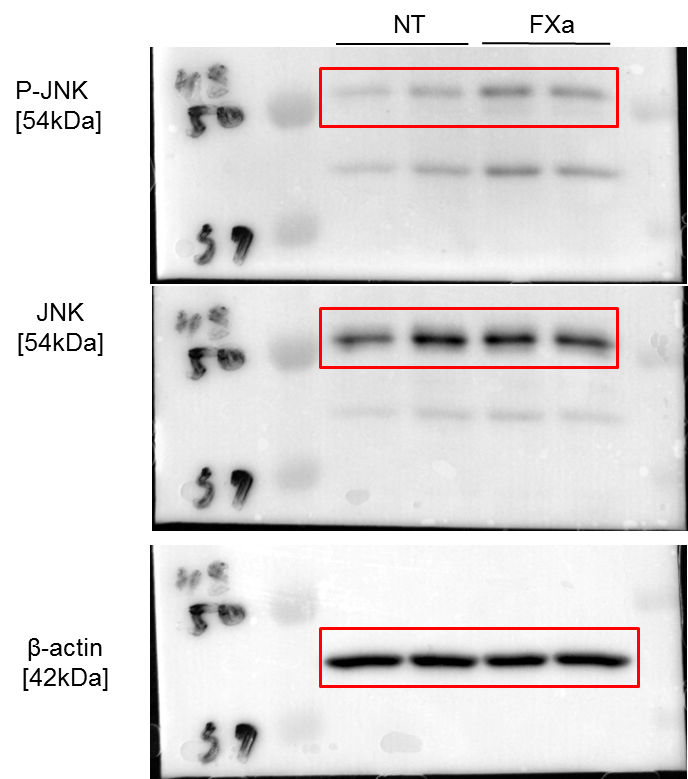


**Supplementary Figure 3. Full-length blot of Figure 3A**

The original full-length immunoblots related to Figure 3A are presented. The top or middle membrane was incubated with anti-phosphorylated JNK antibody and anti-total JNK antibody, respectively. The bottom membrane was incubated with anti-β-actin antibody.


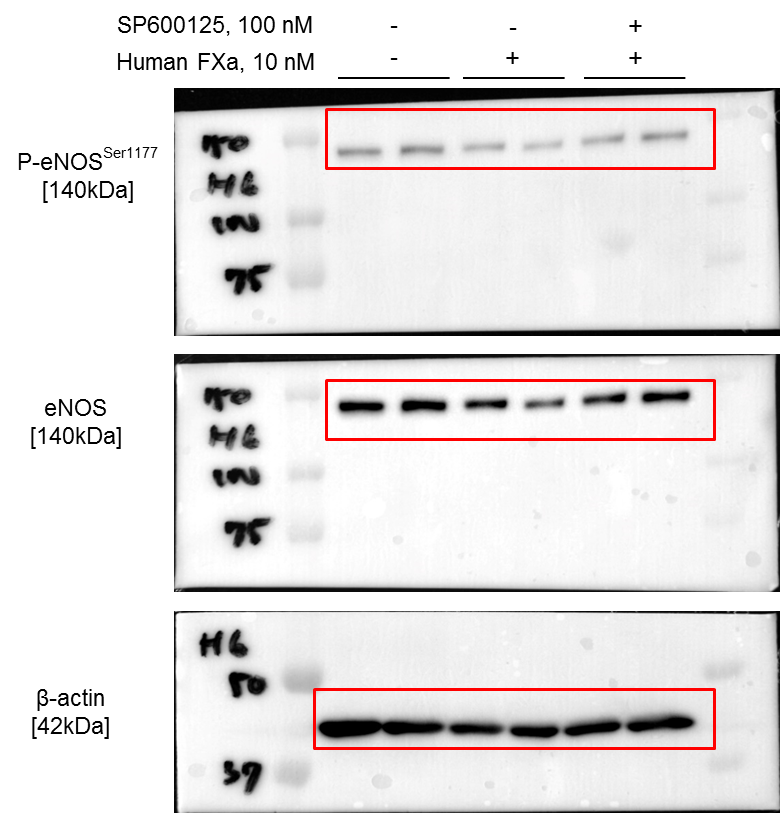


**Supplementary Figure 4. Full-length blot of Figure 3B**

The original full-length immunoblots related to Figure 3B are presented. The top or middle membrane was incubated with anti-phosphorylated eNOS^Ser1177^ antibody or anti-total eNOS antibody, respectively. The bottom membrane was incubated with anti-β-actin antibody.

**
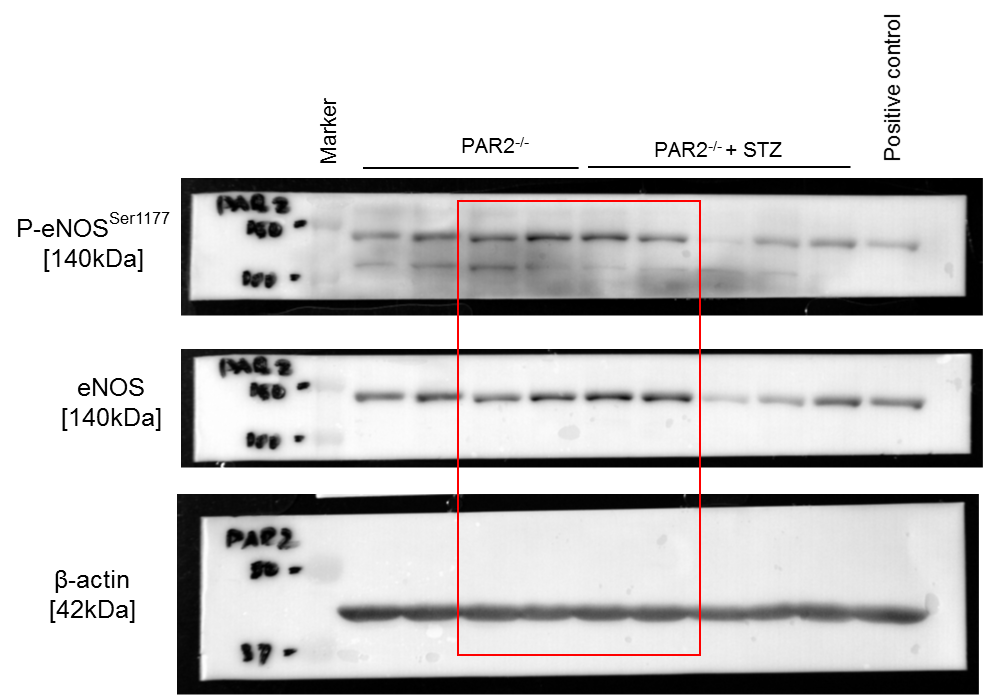
**

**Supplementary Figure 5. Full-length blot of Figure 4C**

The original full-length immunoblots related to Figure 4C are presented. The top or middle membrane was incubated with anti-phosphorylated eNOS^Ser1177^ antibody or anti-total eNOS antibody, respectively. The bottom membrane was incubated with anti-β-actin antibody.


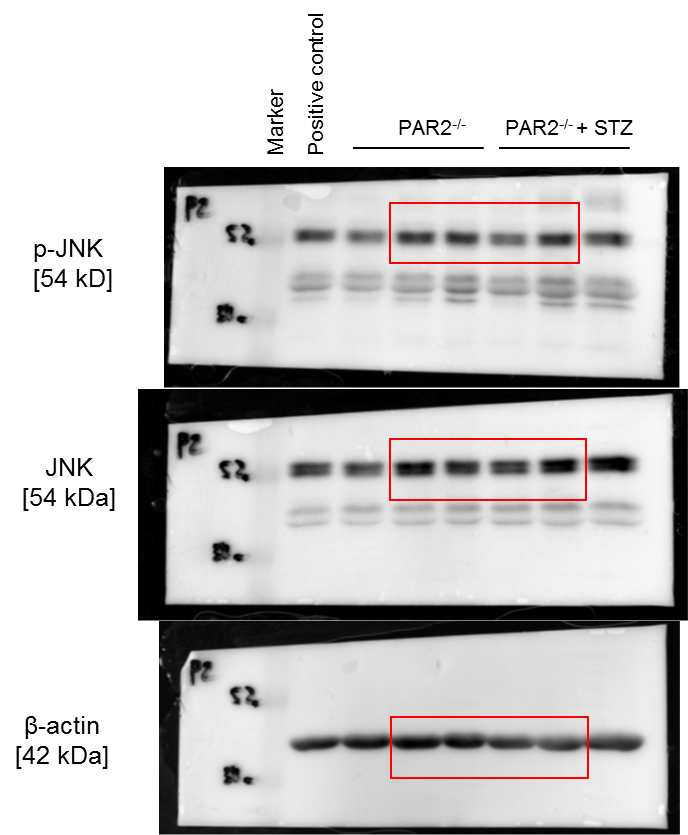


**Supplementary Figure 6. Full-length blot of Figure 4D**

The original full-length immunoblots related to Figure 4D are presented. The top or middle membrane was incubated with anti-phosphorylated JNK antibody or anti-total JNK antibody, respectively. The bottom membrane was incubated with anti-β-actin antibody.
